# Supplementary material for: Prevalence of the Os Supranaviculare: A Systematic Review with Meta-Analysis
Source: J Clin Med. 2025 Aug 22;14(17):5934. doi: 10.3390/jcm14175934 (PMC12429437; doi:10.3390/jcm14175934)
Supplement: Supplementary file 1 [file jcm-14-05934-s001.zip › Supplementary Material S2.pdf]

Supplementary Material S2. Detailed search strategy

The following search terms were employed: "os supranaviculare" OR "supranavicular" OR os talonaviculare dorsale OR os talonavicular dorsal OR astralgo scaphoid ossicle OR pirie bone OR "accessory ossicle". The search techniques for the databases followed the same general design, taking into account the specific syntactic needs of the search engine. All of the records identified up to 20th of May, 2025.

Database	Search terms	Results
Pubmed/Medline	"os supranaviculare" OR "supranavicular" OR os talonaviculare dorsale OR os talonavicular dorsal OR astralgo scaphoid ossicle OR pirie bone OR "accessory ossicle"	519
Embase	'os supranaviculare' OR 'supranavicular' OR 'os talonaviculare dorsale' OR (os AND talonaviculare AND dorsale) OR 'os talonavicular dorsal' OR (os AND talonavicular AND dorsal) OR 'astralgo scaphoid ossicle' OR (astralgo AND scaphoid AND ('ossicle'/exp OR ossicle)) OR 'pirie bone' OR (pirie AND ('bone'/exp OR bone)) OR 'accessory ossicle'	3813
ScienceDirect	"os supranaviculare" OR "supranavicular" OR os talonaviculare dorsale OR os talonavicular dorsal OR astralgo scaphoid ossicle OR pirie bone OR "accessory ossicle"	760
Scopus	os supranaviculare OR supranavicular bone	587
