# Supplementary material for: Prevalence of the Os Supranaviculare: A Systematic Review with Meta-Analysis
Source: J Clin Med. 2025 Aug 22;14(17):5934. doi: 10.3390/jcm14175934 (PMC12429437; doi:10.3390/jcm14175934)

Supplementary material S3. Stratified forest plots for each subgroup category.

A. Males

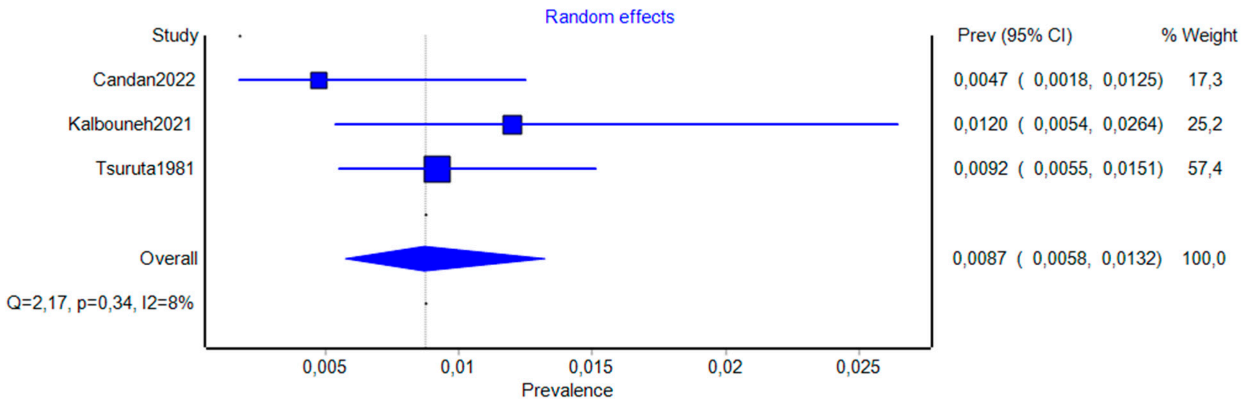

B. Females

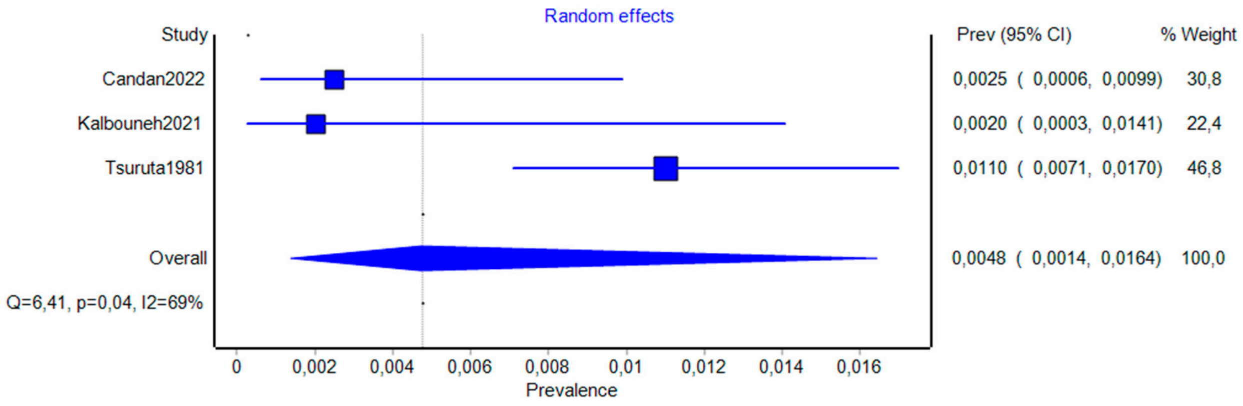

C. Asia

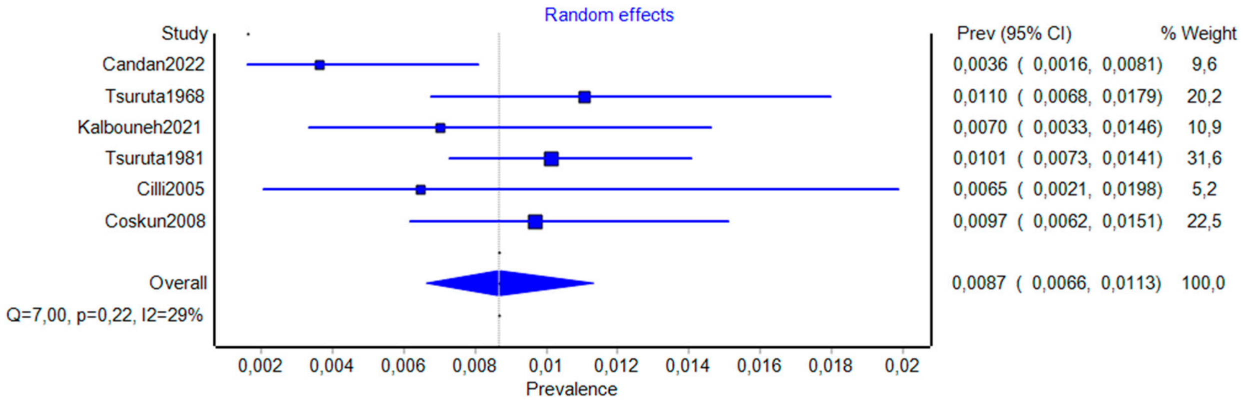

D. Europe

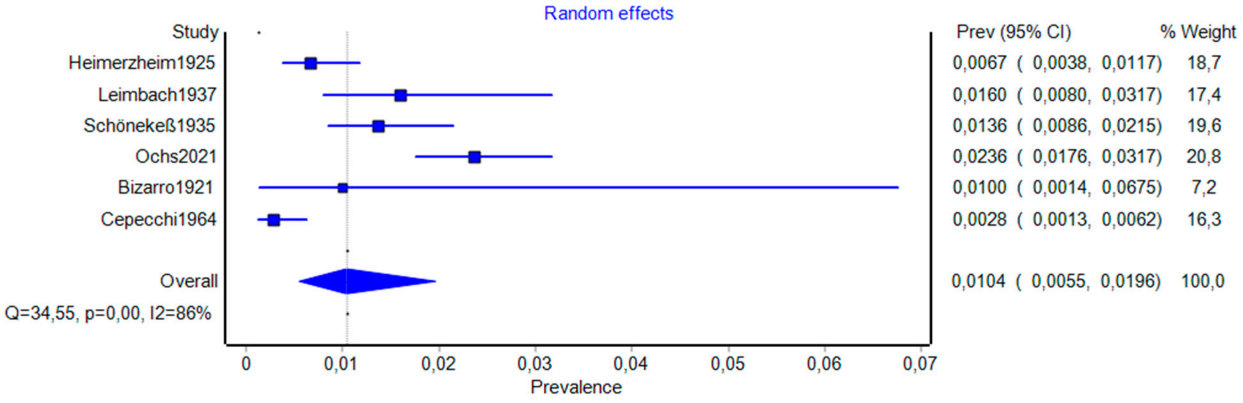

E. Insufficient sample size (<1521 feet)

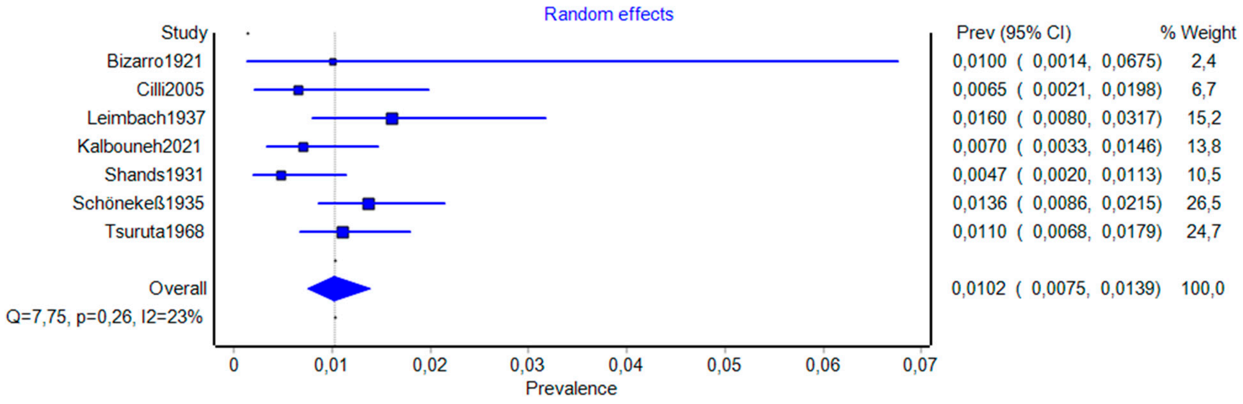

F. Sufficient sample size (>1521 feet)

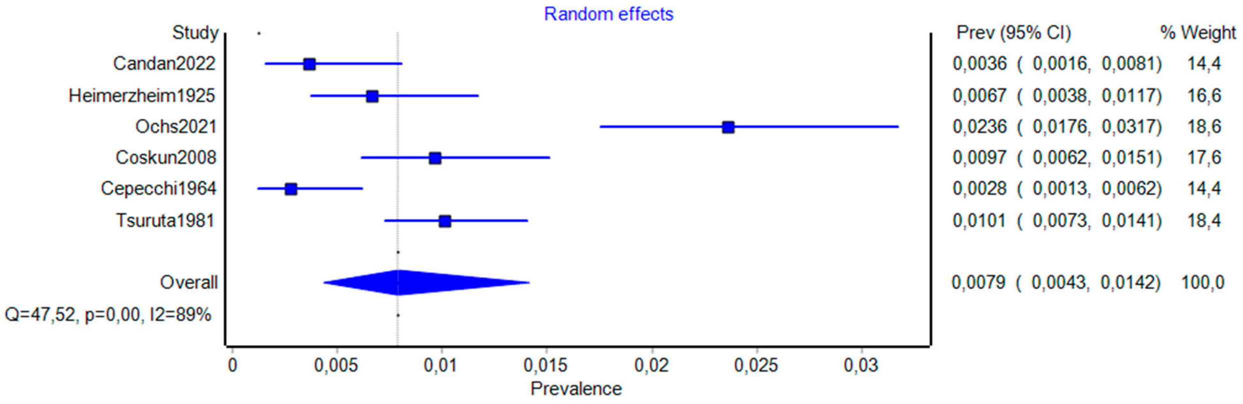

Supplement: Supplementary file 1 [file jcm-14-05934-s001.zip › Supplementary material S3.pdf]
